# Angiodysplasia as a rare cause of acute hematochezia in a 33-year-old with vascular risk factors: a case report

**DOI:** 10.3389/fmed.2026.1829616

**Published:** 2026-05-19

**Authors:** Lin Shi, Jian Lu, Run Liu, Shaohua Sun, Junli Wang, Zhengrong Guo, Yulan Liu

**Affiliations:** 1 Department of Gastroenterology, Shijiazhuang People’s Hospital, Shijiazhuang, Hebei, China; 2 Department of Gastroenterology, Peking University People’s Hospital, Beijing, China

**Keywords:** angiodysplasia, gastrointestinal endoscopy, hypertension, rectal hemorrhage, titanium clips

## Abstract

**Background:**

Rectal angiodysplasia is a vascular malformation typically associated with the elderly. It is an exceedingly rare cause of acute lower gastrointestinal bleeding (LGIB) in young adults. We present a unique case of massive hematochezia in a 33-year-old, potentially accelerated by long-standing vascular stress.

**Case presentation:**

A 33-year-old male with a history of chronic alcohol use and poorly controlled hypertension presented with acute, massive hematochezia and symptomatic anemia. Physical examination and initial labs may indicate significant blood loss. Urgent colonoscopy revealed multiple punctate, actively bleeding angiodysplastic lesions localized strictly to the rectum. Given the focal nature of the lesions, hemostasis was achieved using mechanical endoscopic titanium clips. No recurrent bleeding occurred during 6 months of follow-up.

**Conclusion:**

This single-case observation suggests that angiodysplasia may be considered in differential diagnosis for LGIB even in younger patients, particularly those with systemic vascular risk factors like hypertension and chronic alcohol consumption. Furthermore, it may indicate that mechanical clipping is a highly effective, targeted alternative to thermal ablation for localized rectal vascular lesions.

## 1 Introduction

Gastrointestinal angiodysplasia (GIAD) is a well-characterized vascular malformation defined by the presence of dilated, tortuous, and thin-walled vessels within the mucosal and submucosal layers (1). While GIAD is a leading cause of lower gastrointestinal bleeding (LGIB), it is traditionally viewed as a degenerative condition of aging, with a peak prevalence in patients over 60 years of age (2). Pathophysiologically, these lesions are most frequently localized to the cecum and ascending colon (3, 4), where high wall tension—governed by LaPlace’s Law—is thought to induce chronic, low-grade mucosal ischemia and subsequent vascular ectasia.

The presentation of GIAD in the third decade of life is clinically atypical and warrants a high index of suspicion for underlying predatory factors (5). Furthermore, isolated rectal involvement is significantly less common than its proximal counterparts, often leading to diagnostic delays or misidentification as internal hemorrhoids or proctitis (6–8). In younger cohorts, the early manifestation of these lesions raises the possibility of an accelerated vascular remodeling process, potentially driven by chronic endothelial shear stress from systemic hypertension or the mucosal toxic effects of chronic alcohol consumption (9, 10).

We report a rare case of acute, massive hematochezia originating from rectal angiodysplasia in a 33-year-old male with poorly controlled hypertension and chronic alcohol use. This report aims to highlight the necessity of including vascular malformations in the differential diagnosis of LGIB in young adults with systemic risk factors and to describe the efficacy of targeted mechanical hemostasis in focal rectal lesions (11, 12). This case report was prepared in accordance with the CARE guidelines (13).

## 2 Case presentation

A 33-year-old male presented to the emergency department with a 12-h history of acute, painless hematochezia. He reported 7–8 episodes of bright red stools (∼100 mL per episode) prior to admission, associated with progressive fatigue and mild abdominal distension. His medical history was significant for a 10-year history of poorly controlled hypertension, with home readings frequently reaching 200/150 mmHg. Antihypertensive therapy (nifedipine CR 30 mg/day) was poorly managed with significant non-adherence. Additional risk factors included untreated hyperlipidemia, a 10-year smoking history (20 cigarettes/day), and chronic daily alcohol consumption (50 g ethanol). The patient had no history of liver cirrhosis or chronic kidney disease, and no personal or family of bleeding disorders. There was no family history of hereditary hemorrhagic telangiectasia (HHT) or other vascular disorders, and the patient denied recent NSAID, anticoagulant, herbal supplement, or over-the-counter medication use.

Upon admission, the patient was hypertensive (169/104 mmHg) and tachycardic (102 bpm). Physical examination revealed no stigmata of chronic liver disease (e.g., caput medusae, spider angiomata, or splenomegaly). Initial laboratory investigations showed a hemoglobin (Hb) of 124.3 g/L, blood urea nitrogen(BUN) 7.3 mmol/L, creatinine(Cr) 72 μmol/L, estimated glomerular filtration rate(eGFR) > 90 ml/min/1.73 m^2^,normal coagulation parameters (INR 1.0; platelets 210 × 10^9^/L),and normal liver function tests (ALT 38 U/L,AST 22U/L, total bilirubin 11.7 μmol/L, albumin 40.2 g/L). Chest, abdominal and pelvic computed tomography (CT) was completed. The diagnostic findings are as follows: Coronary artery atherosclerosis, Heterogeneous fatty liver, Cholestasis, Left renal cystic lesion, Tiny calculus of the left kidney, Blurred margin of both kidneys, Thickened appendix, Prostatic calcification. Several hours post-admission, the patient experienced a catastrophic episode of hematochezia totaling approximately 1,000 mL of dark red blood and clots. Serial laboratory monitoring suggested a rapid decline in Hb from 124.3 to 78.4 g/L within 24 h. Following this precipitous Hb decline, the patient became hemodynamically unstable with persistent tachycardia and hypotension, necessitating the transfusion of 7 units of leukocyte-reduced packed red blood cells.

**FIGURE 1:**
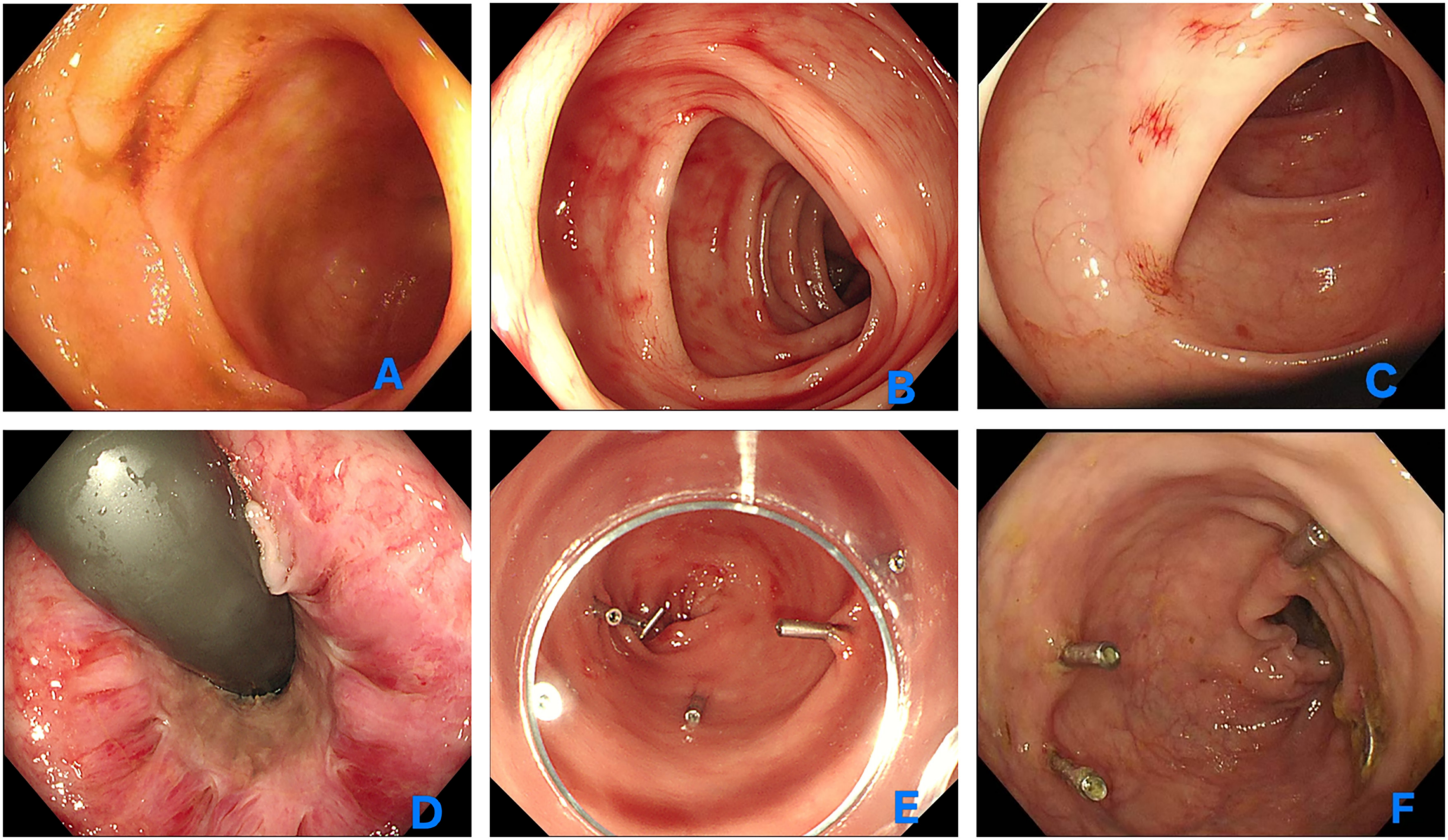
Endoscopic findings and hemostatic management of rectal angiodysplasia. **(A)** Colonoscopic view of the terminal ileum showing clear without evidence of blood or active bleeding. **(B)** Endoscopic image of the rectum demonstrating multiple punctate, actively oozing vascular lesions consistent with angiodysplasia. **(C)** High-definition magnification of a rectal lesion revealing a characteristic “fern-like” or stellate vascular pattern, suggestive of bleeding angiodysplasia. **(D)** Grade I internal hemorrhoids observed in the rectum without signs of recent or active hemorrhage. **(E)** Successful deployment of eight 11-mm mechanical titanium clips over the focal rectal angiodysplastic lesions, resulting in immediate hemostasis. **(F)** Follow-up colonoscopy performed 5 days post-procedure showing residual erythematous vascular ectasias in the rectum with no evidence of re-bleeding.

Given the hemodynamic shift and precipitous Hb drop, an emergency colonoscopy was performed without bowel preparation. The ileocecal junction was reached at 90 cm from the anal verge, the ileocecal valve presented with a normal orifice, and the terminal ileum was intubated for approximately 10 cm through the valve, with no bloody substance observed in the terminal ileum (Figure 1A). Using a water-jet irrigation system, the entire colon were systematically cleared of blood and debris; no proximal bleeding source, ulcers, or diverticula were identified. In the rectum, however, multiple distinct, punctate, actively oozing vascular lesions were identified (Figure 1B). High-definition magnification of the rectal mucosa further suggested the characteristic “fern-like” or stellate pattern of actively bleeding angiodysplasia (Figure 1C). While Grade I internal hemorrhoids were present, they showed no signs of recent or active hemorrhage (Figure 1D). No findings of colitis were noted in the rectum or left colon. To achieve hemostasis, eight 11-mm mechanical titanium clips were applied to the focal rectal lesions, resulting in the immediate cessation of bleeding (Figure 1E).

**FIGURE 2:**
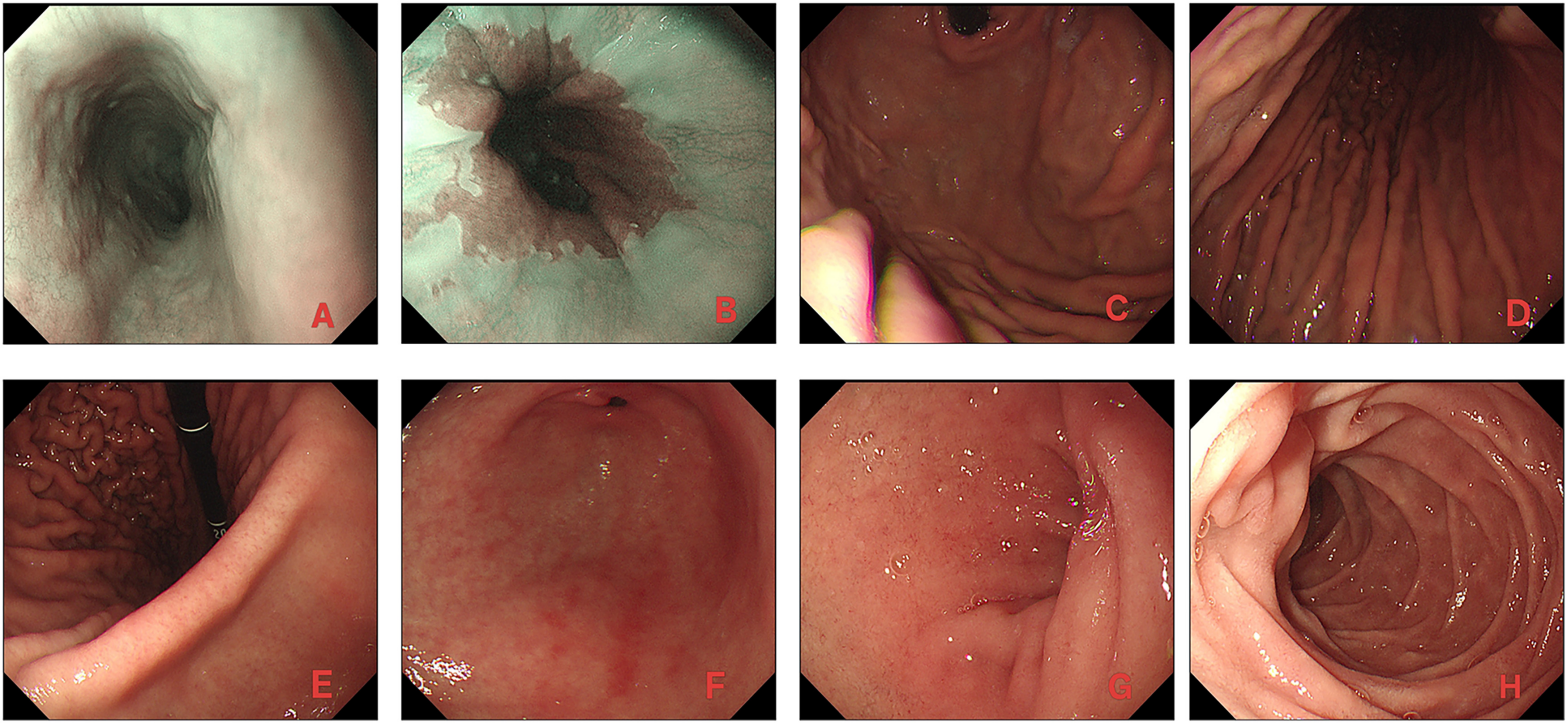
Gastroscopic findings. **(A)** Esophagus. **(B)** Cardia. **(C)** Gastric fundus. **(D)** Gastric body. **(E)** Gastric angulus. **(F)** Gastric antrum. **(G)** Duodenal bulb. **(H)** Second portion of duodenum.

**FIGURE 3:**
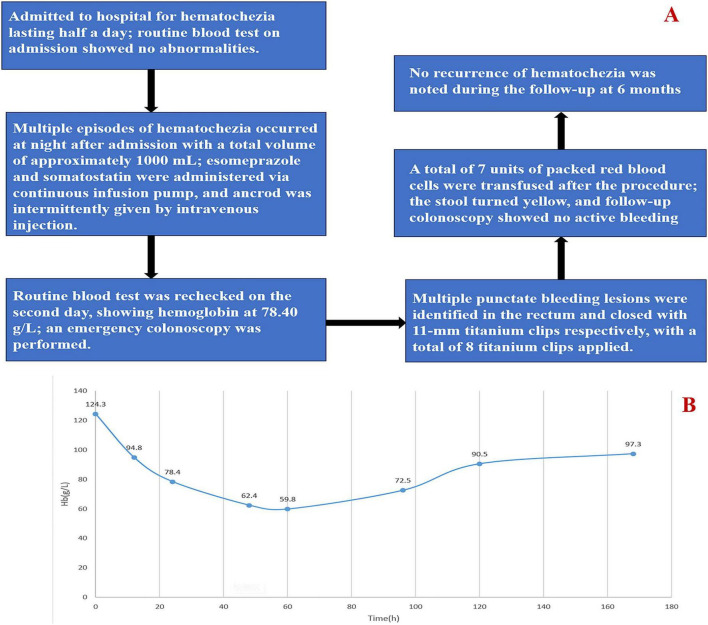
Clinical timeline and hemoglobin trend during the hospital course. **(A)** Clinical events and interventions from presentation to 6-month follow-up. **(B)** Hemoglobin vs. time graph.

The patient’s Hb stabilized post-procedure, and he remained asymptomatic for the remainder of his hospital stay. A follow-up colonoscopy performed 5 days later suggested the presence of residual erythematous vascular ectasias in the rectum without signs of re-bleeding (Figure 1F). Gastroscopy was performed simultaneously, revealing chronic gastritis without other lesions, which rules out upper gastrointestinal bleeding (Figure 2). After clinical stabilization, contrast-enhanced abdominal CT was performed, which showed no newly developed lesions and no additional abnormal findings compared with unenhanced CT. We emphasized to the patient the importance of taking nifedipine controlled-release tablets regularly, and informed him that he must keep his blood pressure below 140/90 mmHg to reduce the risk of rebleeding. The patient was discharged with a reinforced antihypertensive regimen and alcohol cessation counseling. At the 6-month follow-up, the patient reported no recurrence of hematochezia, and his Hb remained stable at 132 g/L. Figure 3 summarizes the sequential clinical events and therapeutic interventions throughout the patient’s hospital course, alongside the trend of hemoglobin levels over time.

## 3 Discussion

Gastrointestinal angiodysplasia (GIAD) is characterized by ectatic, thin-walled submucosal vessels that lack a smooth muscle layer, making them highly susceptible to rupture under mechanical stress. While traditionally viewed as a degenerative disease of the elderly (14)—driven by chronic, intermittent, low-grade venous obstruction—its manifestation in a 33-year-old suggests an accelerated vascular aging phenotype.

Table 1 summarizes selected case reports and small case series of gastrointestinal angiodysplasia in patients ≤ 50 years of age published over the past 15 years. The present case, involving a 33-year-old male with isolated rectal involvement, is notable both for its rectal localization (documented in only a subset of younger cases) and for its occurrence in the absence of congenital syndromes, von Willebrand disease, or identifiable inherited vascular disorders. The present case differs from most previously reported young-patient cases in its isolated rectal localization and its occurrence in the setting of poorly controlled essential hypertension without congenital or hematologic predisposition. This constellation of findings reinforces the concept that systemic vascular stress alone, in the absence of inherited disorders, may suffice to produce clinically significant angiodysplasia in younger adults—a concept with direct implications for risk stratification and follow-up planning in similar patients.

**TABLE 1:** Summary of reported cases of gastrointestinal angiodysplasia in young patients.

References	Case number	Patient age/sex	Lesion site	Risk factors	Treatment
Nilojan et al. (26)	1	42, M	Ileocecal junction	Type 2 DM	Superselective transcatheter glue embolization
Dabora et al. (12)	1	23, M	Jejunum (40 cm from DJ flexure)	None	Segmental jejunal resection + anastomosis
Gumaa Albashari et al. (11)	1	19, M	Diffuse: jejunum to sigmoid colon	Possible undiagnosed bleeding disorder(positive family history)	Multiple endoscopic therapies + laparotomy with intraoperative endoscopy + 70 cm small bowel resection
Brockway et al. (27)	1	23, M	Ascending and transverse colon (large AVM)	Tobacco, alcohol, occasional cocaine use	Colonoscopic clip + surgical vessel ligation + embolization
Kimpton et al. (28)	1	10, F	Jejunum (100 cm from DJ flexure)	None	Segmental jejunal resection (30 cm)
Olokoba et al. (29)	1	30, F	Sigmoid colon (25 cm from anal verge)	None	Blood transfusion;colonoscopy after stabilization
Goel et al. (30)	1	20, M	Proximal jejunum	Malrotation of bowel	Segmental jejunal resection

M, Male; F, Female; DJ, duodenojejunal; AVM, arteriovenous malformation;DM, diabetes mellitus.

### 3.1 Co-occurrence of hypertension and ethanol exposure

In this patient, the 10-year history of “malignant-range” hypertension (200/150 mmHg) likely acted as a primary driver of endothelial shear stress (15, 16). We hypothesize that these repetitive pressure spikes induced compensatory but maladaptive remodeling of the rectal microvasculature. Notably, a recent retrospective study (17) found that while anticoagulant use significantly increased bleeding risk in angiodysplasia, hypertension alone was not independently associated with bleeding, suggesting that hypertension may contribute primarily to lesion formation rather than to the acute precipitation of hemorrhage. This was likely compounded by chronic ethanol consumption (18), which acts as a systemic vasodilator while simultaneously inducing oxidative damage to the vascular basement membrane.

From a mechanistic perspective, chronic hypertension generates sustained endothelial shear stress, which may promote conformational changes in von Willebrand factor (vWF) multimers, rendering them susceptible to proteolytic cleavage by ADAMTS13. The resulting reduction in high-molecular-weight vWF multimers is thought to disinhibit vascular endothelial growth factor (VEGF) signaling, thereby facilitating aberrant angiogenesis and the formation of angiodysplastic lesions (19). Additionally, VEGF and basic fibroblast growth factor have been shown to be overexpressed in human colonic angiodysplasia tissue, further supporting a pro-angiogenic milieu in these lesions (20). Simultaneously, chronic ethanol exposure induces endothelial barrier dysfunction through oxidative stress-mediated disruption of tight junction proteins and basement membrane integrity. Ethanol activates the renin-angiotensin-aldosterone system, upregulates NADPH oxidase, and increases mitochondrial reactive oxygen species production, leading to reduced nitric oxide bioavailability and direct endothelial injury (21). The co-occurrence of these two stressors-hypertension-driven angiogenic signaling and alcohol-induced endothelial fragility-may create a synergistic environment favoring the premature development and rupture of angiodysplastic lesions. However, given the single-case design (*n* = 1) and the presence of multiple coexisting factors (hypertension, alcohol, smoking, hyperlipidemia), the independent contribution of any single variable cannot be determined. These observations should be considered hypothesis-generating rather than definitive.

### 3.2 Diagnostic prioritization of angiodysplasia

The presence of Grade I internal hemorrhoids initially raised the possibility of hemorrhoidal bleeding; however, the absence of stigmata of recent hemorrhage on the hemorrhoidal cushions, coupled with the characteristic “fern-like” endoscopic pattern and the hemodynamic significance of blood loss (Hb decline from 124.3 to 78.4 g/L with transfusion requirement), prioritized angiodysplasia as the primary diagnosis. This case highlights the importance of systematic rectal mucosal inspection rather than attributing massive hematochezia to incidentally noted hemorrhoids.

### 3.3 Therapeutic rationale

While Argon Plasma Coagulation (APC) is the conventional “workhorse” for GIAD (22), we opted for mechanical clipping. In the rectum, focal punctate lesions allow for the precise application of clips, which provides immediate primary hemostasis without the risk of deep thermal injury or transmural necrosis—a particularly relevant concern when multiple lesions are clustered in a small area. While APC is generally safe in the rectum, the presence of ongoing oozing and a blood-filled field can reduce its efficacy due to the “heat sink” effect of flowing blood. Clips provided definitive, visually confirmed hemostasis without these limitations.

Furthermore,a recent study by Arimoto et al. described a cold snare technique with clipping for colorectal angioectasia, which enable direct visualization and mechanical occlusion of the feeding vessel (23). While our case employed clipping alone, both approaches share a common hemostatic principle—targeted mechanical interruption of the submucosal feeding vessel—and may represent an evolving paradigm for the endoscopic management of focal angiodysplastic lesions.

### 3.4 Differential diagnoses and exclusions

In young patients with acute massive lower gastrointestinal bleeding, key differential diagnoses include upper gastrointestinal bleeding, small bowel bleeding, Meckel diverticulum, Dieulafoy lesion, neoplasia, ulcerative lesions, and hemorrhoids. In the present case, upper gastrointestinal bleeding was excluded by gastroscopy and BUN/Cr ratio, small bowel sources were excluded by complete endoscopic examination of the terminal ileum, and Meckel diverticulum, Dieulafoy lesion were all ruled out endoscopically, small bowel neoplasia was further evaluated by contrast-enhanced computed tomography; however, it should be acknowledged that conventional contrast-enhanced CT has limited sensitivity for detecting flat mucosal vascular lesions such as small bowel angiodysplasia or small submucosal neoplasms compared with dedicated CT enterography, which was not performed in this case. Despite this limitation, the clear proximal-to-distal blood density gradient with maximal blood in the rectum, the absence of small bowel abnormalities on contrast-enhanced CT, and the sustained bleeding-free status at 6-month follow-up collectively argue against a missed proximal small bowel source. In the emergency setting of hemodynamic instability, urgent colonoscopy with terminal ileum intubation and standard contrast-enhanced CT constituted a pragmatic diagnostic approach. Although internal hemorrhoids were present, they exhibited no evidence of active bleeding. Accordingly, the definite bleeding source was suggested as actively bleeding rectal angiodysplasia.

### 3.5 Discrepancy between lesion appearance and bleeding severity

Although the angiodysplastic lesions visualized during colonoscopy appeared punctate and non-pulsatile, two factors may reconcile this finding with the substantial hemoglobin decline observed. First, the cumulative blood loss from multiple synchronous actively oozing lesions can account for the rapid reduction in hemoglobin over the initial 24 h. Second, as hemorrhage progressed, the patient’s systemic blood pressure declined progressively, leading to reduced perfusion pressure at the lesional level. This hemodynamic autoregulatory effect likely attenuated the intensity of bleeding, such that by the time of urgent colonoscopy the lesions manifested as punctate oozing rather than pulsatile arterial hemorrhage.

### 3.6 Limitations

This report has significant methodological limitations inherent to its design as a single-case report (*n* = 1). No statistical analysis was performed, and no causal inference is possible. The findings are not generalizable to broader populations. The patient presented with multiple coexisting vascular risk factors, creating a non-identifiable structure in which the independent effect of any single factor cannot be disentangled. Measurements such as estimated blood loss are semi-quantitative and subject to error. Additionally, inherent limitations such as publication bias and selection bias must be considered when interpreting isolated case observations, as previously discussed in the methodological literature (24). The external validity of this observation is negligible, and the report should be viewed as hypothesis-generating, low-level evidence within the GRADE framework (25).

## 4 Conclusion

This case suggests that rectal angiodysplasia, though rare in the young, should be included in the differential diagnosis of massive LGIB in patients with systemic vascular stressors. The successful 6-month outcome supports a targeted mechanical approach to hemostasis. Clinicians should remain vigilant for “elderly” pathologies in younger cohorts when significant cardiovascular risk factors are present. Despite the inherent limitations of a single-case design, this report provides a clinically actionable reminder that the differential diagnosis for massive LGIB in young adults with poorly controlled hypertension should extend beyond hemorrhoidal bleeding and inflammatory conditions to include rectal vascular malformations, as timely endoscopic recognition can avert diagnostic delays and unnecessary interventions.

## Data Availability

The original contributions presented in this study are included in this article/supplementary material, further inquiries can be directed to the corresponding authors.
